# Negligible effect of vitamin D supplementation on exacerbation in patients with chronic obstructive pulmonary disease: meta-analysis

**DOI:** 10.11613/BM.2023.030703

**Published:** 2023-10-15

**Authors:** Ye Hua, Ting Jiang, Jiangyi Feng, Mi Zou

**Affiliations:** 1 Department of general surgery, Chongqing Emergency Medical Center, Chongqing University Central Hospital, Chongqing, China; 2 Department of blood transfusion, Chongqing Emergency Medical Center, Chongqing University Central Hospital, Chongqing, China; 3 Respiratory department, The First branch of the first affiliated hospital of Chongqing Medical University, Chongqing, China

**Keywords:** chronic obstructive pulmonary disease, vitamin D deficiency, disease progression, meta-analysis

## Abstract

**Introduction:**

The focus of this meta-analysis was how vitamin D supplementation influences exacerbations in patients with chronic obstructive pulmonary disease (COPD) and vitamin D deficiency (VDD).

**Materials and methods:**

Cochrane Library, Web of Science, Embase, and PubMed databases have been systematically searched in an attempt to collect randomized controlled trials related to vitamin D supplementation in COPD patients with VDD published in English available by July 2022. Primary outcome indicators included the mean number of exacerbation and rate of exacerbation. Secondary outcome indicators included forced expiratory volume in the first second (FEV1), FEV1/forced vital capacity (FVC) ratio, and serum 25-hydroxyvitamin D (25(OH)D) concentration.

**Results:**

Five studies involving 522 COPD patients with VDD (defined as 25(OH)D < 50 nmol/L) were included, among them 61 were severely deficient in vitamin D (25(OH)D < 25 nmol/L). The results showed that vitamin D supplementation did not decrease the mean number of exacerbation (standardized mean difference (SMD): - 0.10, 95% CI: - 0.29 to 0.09) and the rate of exacerbation (relative risk (RR): 0.89, 95% CI: 0.76 to 1.04, P = 0.179). Also, its effect on FEV1 (SMD: - 0.06, 95% CI: - 0.30 to 0.17) and FEV1/FVC (SMD: -0.10, 95% CI: - 0.48 to 0.27) remained negligible. However, it could increase the serum 25(OH)D concentration (SMD: 2.44, 95 CI%: 2.20 to 2.68, P < 0.001).

**Conclusions:**

The effects of vitamin D supplementation on decreasing exacerbation and improving pulmonary function were not significant.

## Introduction

With relatively high incidence and mortality rates across the globe, chronic obstructive pulmonary disease (COPD) is characterized by long-term respiratory symptoms and incompletely reversible airflow limitation (*1*-*3*). Exacerbations of COPD are defined as acute exacerbations of respiratory symptoms that require additional treatment (*4*). Clinical symptoms of COPD include chronic cough, expectoration, shortness of breath or dyspnoea. Spirometry is the diagnostic standard for COPD, and assessment of airflow limitation is a common pulmonary function test (*5*). Forced expiratory volume in the first second (FEV1) / forced vital capacity (FVC) ratio can evaluate the pulmonary function, which assists in diagnosis and evaluation of COPD progression (*6*). Pathophysiological changes in COPD patients are mainly characterized by ciliary dysfunction, abnormal gas exchange, and airway mucus hypersecretion, among which airway mucus hypersecretion is associated with small airway inflammation (*7*). Reportedly, COPD has comorbidities such as cardiovascular disease, osteoporosis, diabetes mellitus, and hypertension (*8*, *9*). Vitamin D is involved in calcium and phosphorus metabolism as well as immune and inflammation regulation, bearing a close relation with chronic diseases including autoimmune diseases, diabetes, cardiovascular diseases, and respiratory diseases (*10*, *11*). Apart from that, serum 25-hydroxyvitamin D (25(OH)D) concentration is confirmed to be associated with a certain magnitude of vitamin D deficiency (VDD), a common situation among COPD patients (*12*). Vitamin D deficiency in COPD patients is also found to be associated with the reduced resistance of the respiratory mucosa, reduced respiratory immune function, and increased risk of respiratory tract infection (*13*, *14*).

Recent studies have pointed out the essential role of vitamin D in COPD prevention and therapy, which improves pulmonary function to control acute attack frequency and improve prognosis (*15*). Relevant reports also suggested that supplementation of vitamin D can reduce the exacerbation rate of COPD patients with VDD (*16*, *17*). Nevertheless, Jolliffe *et al.* observed a negligible effect of vitamin D3 on the exacerbation rate of COPD patients (*18*). Similarly, some scholars also put forward that vitamin D supplementation has little influence on the exacerbation rate of COPD patients with VDD (*19*, *20*). Though two existing meta-analyses have confirmed that vitamin D can increase FEV1 and FEV1/FVC ratio and reduce the number of exacerbation rate, a separate analysis targeting COPD patients with VDD is yet to be performed (*15*, *21*).

Therefore, in this study, we conducted a meta-analysis to investigate the effect of vitamin D supplementation on COPD patients with VDD, with the aim of providing reference for clinical treatment.

## Materials and methods

### Literature search

Embase, Web of Science, PubMed, and Cochrane Library databases had been systematically searched for English studies on vitamin D treatment in COPD patients with VDD available from the establishment of the databases to July 2022. Search keywords included “vitamin D”, “vitamin D deficiency”, “exacerbation”, and “chronic obstructive pulmonary disease”. References of included studies were screened manually for possible studies that meet the inclusion criteria.

### Inclusion criteria

The inclusion criteria were: 1) patients aged 40-80 years; 2) randomized controlled trials (RCT); 3) patients who met the diagnostic criteria stated in Guidelines for the diagnosis and management of chronic obstructive pulmonary disease (revised version 2021); 4) serum 25(OH)D concentration was adopted as a laboratory marker for the diagnosis of VDD; 25(OH)D < 50 nmol/L indicated deficiency; 25(OH)D < 25 nmol/L indicated severe deficiency; 5) patients were randomly assigned to receive vitamin D (experimental group) or placebo (control group) (*22*, *23*).

### Exclusion criteria

Exclusion criteria were: 1) abstract, review, pathology report, editorial, or expert opinion only; 2) repeatedly published research or studies (similarity of research titles, high overall text duplication rate, and same first author); 3) results cannot be obtained through calculation; 4) animal experimental studies; 5) unclear grouping; 6) patients with a history of hypercalcemia, sarcoidosis, or active cancer.

### Data extraction and quality assessment

After reading the full text, two investigators performed data extraction independently following the inclusion and exclusion criteria. Disagreements were addressed by a third investigator. Extracted information included the first name of authors, publication time, age, sample size, interventions, treatment time, primary outcome indicators (mean number of exacerbation, exacerbation rate), and secondary outcome indicators (FEV1/FVC ratio, serum 25(OH)D concentration).

Following the guidelines of preferred reporting items for systematic reviews and meta-analyses (PRISMA), data extraction by using the Cochrane risk-of-bias tool, which is widely used to measure the methodological quality of RCT and to assess bias risk for the 5 articles included (*24*, *25*). These studies were evaluated mainly by 7 items from the following 6 domains: selection, performance bias, detection bias, follow-up, reporting bias, and other sources of bias. According to the risk-of-bias criteria, each item is judged as “low risk of bias”, “high risk of bias” and “unclear”.

In the selected studies, vitamin D concentration was determined using either radioimmunoassay or liquid chromatography. The vitamin D measurement can be influenced by factors such as time and location. Only two of the included studies conducted experiments during specific seasons (*16*, *20*). Although vitamin D measurement timing was not explicitly specified, the location was described in each study, with measurements taken in the Netherlands, Iran, and Belgium, respectively.

### Statistical analysis

Meta-analysis was performed by Review Manager 5.4 software and Stata 15.0 software with standardized mean difference (SMD) and mean difference (MD) representing continuous variables, and relative risk (RR) representing dichotomous variables (*26*). The I^2^ statistic was used to evaluate the heterogeneity of the included studies. If I^2^ > 50% and P < 0.1, there was statistical heterogeneity among the studies and a random-effects model was used. Otherwise, there was no statistical heterogeneity and a fixed-effects model was used for meta-analysis.

## Results

### Literature search results

**Figure 1:**
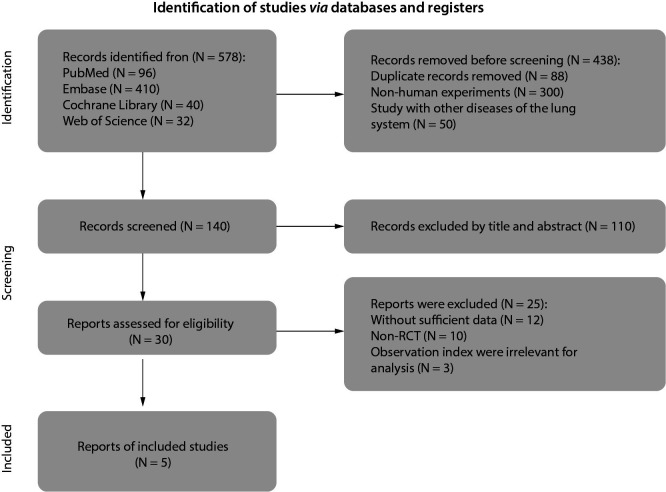
Literature screening process. RCT - randomized controlled trials.

Among 578 studies yielded from the preliminary search, 88 repeatedly published studies, 300 studies with animal experiments, and 50 studies focusing on other pulmonary system diseases were excluded. Studies that deviated from our objective, 110 of them, were excluded after reading the titles and abstracts, and the remaining 30 articles were assessed. A total of 25 review articles and studies that only contained abstracts, conducted non-RCT studies, or had data that cannot be extracted were counted out. Hence, 5 RCT studies that met the threshold were included (*16*, *19*, *20*, *27*, *28*). The screening processes are shown in Figure 1.

### Characteristics and quality assessment of included studies

**Figure 2:**
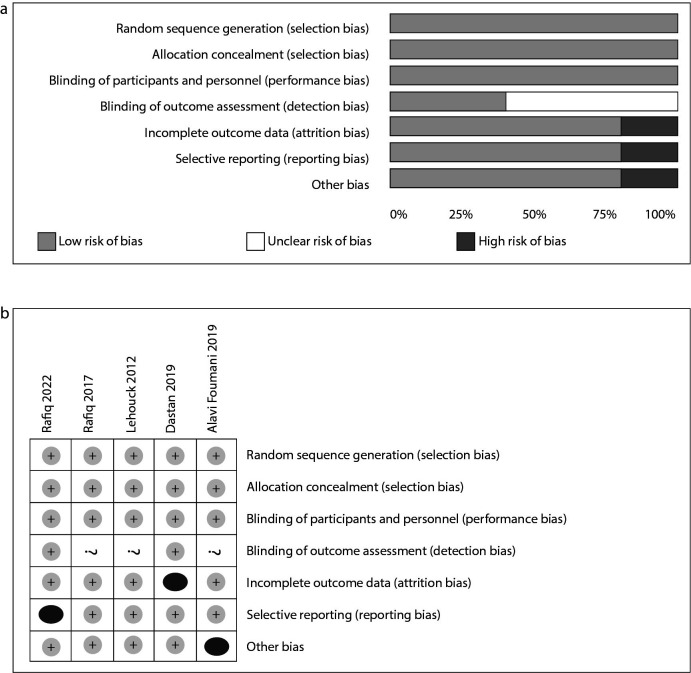
Risk of bias graph (a) and summary of bias risk (b).

A total of 522 COPD patients with VDD were included in the 5 selected studies. Among them, 5 articles reported serum 25(OH)D concentration, 2 reported FEV1, 2 reported FEV1/FVC, 4 kept track of the number of exacerbation, and 2 recorded the rate of exacerbation. Table 1 provides the characteristics of included studies. These studies were of high quality as displayed in Figure 2.

**Table 1:** Characteristics of included studies

**Authors**	**Year**	**Treatment time**	**Sample size**	**Cases** **E/C**	**Age (years)**	**Intervention**	**Outcome**
Rafiq *et al.*	2022	12-month	155	74/81	E: 65 ± 9 C: 67 ± 9	E: 16,800 IU week of vitamin D C: Placebo	①④⑤
Dastan *et al.*	2019	6-day	70	35/35	E: 64.42 ± 7.58 C: 63.24 ± 8.41	E: Vitamin D C: Placebo	①
Alavi Foumani *et al.*	2019	6-month	65	32/31	E: 67.9 ± 7.9 C: 68.4 ± 7.8	E: 50,000 IU of vitamin D3 C: Placebo	①②③④
Rafiq *et al.*	2017	6-month	50	24/26	E: 64 ± 3.7 C: 61 ± 5.9	E: 1200 IU daily of vitamin D3 C: Placebo	①②③④
Lehouck *et al.*	2012	12-month	182	91/91	E: 68 ± 9 C: 68 ± 8	E: 100,000 IU vitamin D of four week C: Placebo	①②④⑤
① - 25(OH)D: 25-hydroxyvitamin D. ② - FEV1 - forced expiratory volume in the first second. ③ - FEV1/FVC - forced expiratory volume in the first second / forced vital capacity. ④ - mean exacerbation number. ⑤ - exacerbation rate. E - experimental group. C - control group.							

### Results of the meta-analysis

#### Mean number of exacerbations

**Figure 3:**
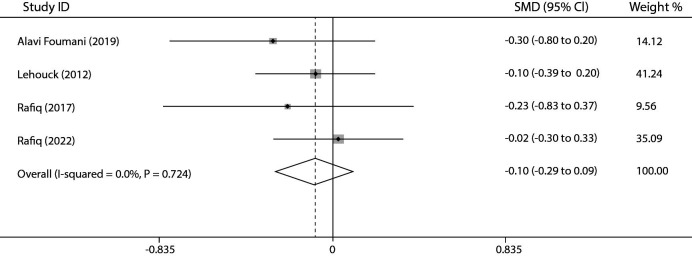
Pooled analysis of the mean number of exacerbation before and after intervention. CI - confidence interval. SMD - standardized mean difference. CI - confidence interval.

The number of exacerbations available in 4 studies was included for meta-analysis, with 452 patients, among them 221 were in the experimental group and 231 in the control. No heterogeneity was observed from the meta-analysis results (I^2^ = 0%, P = 0.724). Subsequently, the fixed-effects model confirmed the negligible effect of vitamin treatment on decreasing the mean number of exacerbation (SMD: - 0.10, 95% CI: - 0.29 to 0.09) as shown in Figure 3.

#### Subgroup analysis on the rate of exacerbation

**Figure 4:**
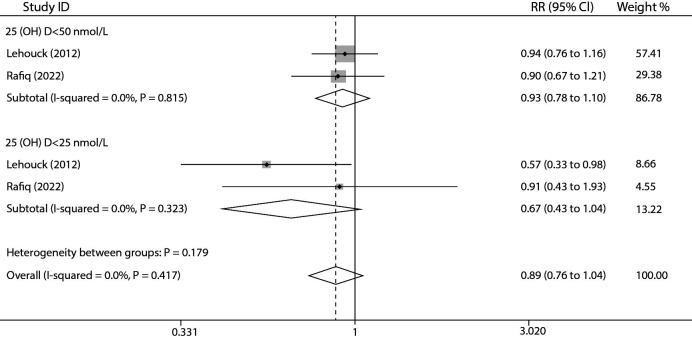
Pooled analysis of exacerbation rate in COPD patients deficient or severely deficient in vitamin D. 25(OH)D - 25-hydroxyvitamin D. RR - relative risk. CI - confidence interval.

The changes in exacerbation rate in COPD patients with VDD and severe VDD were recorded in 2 studies throughout the vitamin D intervention. We grouped the included patient by serum 25-OH-D concentrations with cut-off values of 25 and 50 nmol/L. The fixed-effects model analysis showed (I^2^ = 0%, P = 0.417) that neither patients with VDD (I^2^ = 0%, P = 0.815) nor patients with severe VDD (I^2^ = 0%, P = 0.323) responded with a decrease in exacerbation rate. The pooled results exhibited that vitamin D intervention brought about a negligible decrease in the exacerbation rate of COPD patients with different magnitudes of VDD (RR: 0.89, 95% CI: 0.76 to 1.04, P = 0.179) as shown in Figure 4.

#### FEV1

**Figure 5:**
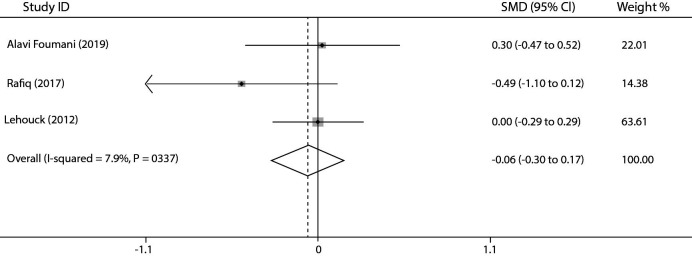
Pooled analysis of FEV1 changes before and after intervention. SMD - standardized mean difference. CI - confidence interval.

Among 3 studies that mentioned FEV1, the number of included patients reached 288, among whom 141 received vitamin D and 147 received placebo. As we included FEV1 changes before and after intervention in the meta-analysis, low heterogeneity was found among the results of the 3 included studies (I^2^ = 7.9%, P = 0.337). As Figure 5 shows, the fixed-effects model displayed no substantial increase in FEV1 among COPD patients administrated with vitamin D (SMD: - 0.06, 95% CI: - 0.30 to 0.17).

#### FEV1/FVC

**Figure 6:**
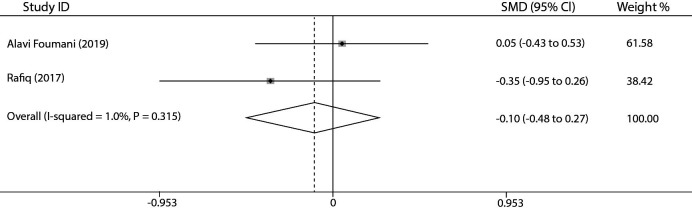
Pooled analysis of FEV1/FVC changes before and after intervention. SMD - standardized mean difference. CI - confidence interval.

Among two studies that mentioned FEV1/FVC, 115 patients were included, and among them 56 received vitamin D and 59 received placebo. As we included FEV1/FVC changes among COPD patients before and after intervention in the meta-analysis, no notable heterogeneity could be observed (I^2^ = 1.0%, P = 0.315). According to the fixed-effects model, vitamin D supplementation brought out no substantial FEV1/FVC increase in COPD patients with VDD (SMD: - 0.10, 95% CI: - 0.48 to 0.27) as shown in Figure 6.

#### Serum 25(OH)D concentration

**Figure 7:**
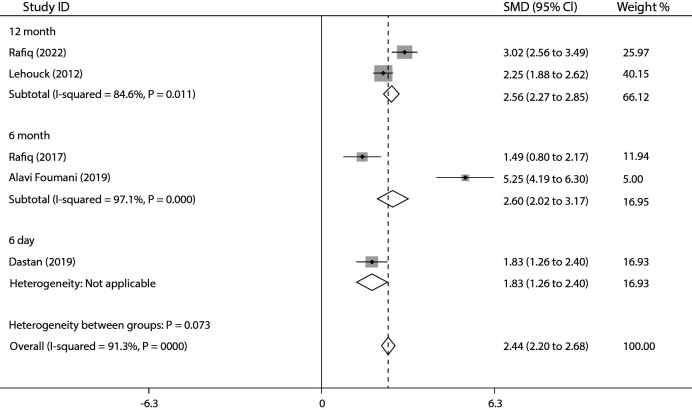
Pooled analysis of serum 25(OH)D concentration changes before and after intervention. SMD - standardized mean difference. CI - confidence interval.

Five studies recorded mean and SMD of serum 25(OH)D concentration changes throughout vitamin D treatment. Two studies showed a visible rise in the serum 25(OH)D concentrations among those who went through 12 months of treatment (SMD: 2.56, 95% CI: 2.27 to 2.85). The other 2 studies with treatment lasting for 6 months suggested that patients had no significant increase in serum 25(OH)D concentrations (SMD: 2.60, 95% CI: 2.02 to 3.17). One study with only 6 days of treatment discovered a significant elevation in serum 25(OH)D concentrations (SMD: 1.83, 95% CI: 1.26 to 2.40). These observations showed a link between vitamin D supplementation and serum 25(OH)D concentration increase in COPD patients with VDD (SMD: 2.44, 95CI%: 2.20 to 2.68, P < 0.001) as shown in Figure 7.

## Discussion

This study conducted a meta-analysis on how vitamin D supplementation influences exacerbation in COPD patients with VDD. The results showed that vitamin D intervention did not significantly improve the number of exacerbation or pulmonary function in patients, but it significantly increased the concentrations of serum 25(OH)D. The positive correlation between serum 25(OH)D concentration and pulmonary function has been confirmed by the extensive exploration of the physiological function of vitamin D (*29*, *30*). Such findings may bear some clinical significance in COPD patients (*28*). Driven by a growing demand to investigate how vitamin D functions in treating COPD, asthma, and other respiratory diseases, a number of promising publications have pointed out the potential use of vitamin D in the treatment of COPD (*31*). In addition, vitamin D supplementation can also treat common COPD comorbidities such as osteoporosis and diabetes mellitus (*32*). Therefore, we conducted the following analysis.

In the present study, the results exhibited that vitamin D intervention brought about a negligible decrease in the exacerbation rate of COPD patients with different magnitudes of VDD, which is in line with the findings of Rafiq *et al.* and Alavi Foumani *et al.* (*19*, *20*). 25-hydroxyvitamin D is considered the best indicator of vitamin D status in the body. Nevertheless, one RCT proved that by supplementing vitamin D, the risk of moderate or severe exacerbation in COPD patients with lower 25(OH)D concentrations can be reduced (*17*). Subsequently, a subgroup analysis on the rate of exacerbation was performed. According to subgroup analysis results, vitamin D supplementation had no effect on decreasing the rate of exacerbation in COPD patients with severe VDD. This is inconsistent with the research that suggested that vitamin D supplementation can reduce the rate of exacerbation in COPD patients with severe VDD (*16*).

Furthermore, this study found that by supplementing vitamin D, no notable improvement in pulmonary function could be seen in COPD patients with VDD. Whereas studies conducted by Martineau *et al.* and Lehouck *et al.*, illustrated that vitamin D supplementation does improve pulmonary function (including FEV1 and FEV1/FVC) of COPD patients (*16*, *17*). Vitamin D deficiency is common in COPD patients and has been associated with reduced pulmonary function and increased risk of exacerbations. The beneficial effects of vitamin D supplementation are supported by another two meta-analyses, which also discovered that vitamin D increases FEV1 and FEV1/FVC in COPD patients (*15*, *21*).

This analysis also uses treatment time as another variable. The results saw a substantial rise in serum 25(OH)D concentrations, regardless of vitamin D supplementation time, which is consistent with previous studies that vitamin D supplementation can increase 25(OH)D concentrations (*16*, *17*, *20*). In addition, due to the immune-regulatory effects of vitamin D, we had intended to investigate the inflammatory biomarkers concentrations, such as C-reactive protein. However, since the results from the included studies were not significant, we did not analyse them. If there are relevant literature reports in the future, we may consider conducting an updated meta-analysis.

Unfortunately, this meta-analysis has some limitations: 1) included COPD patients had different disease severities; 2) insufficient data failed us to perform subgroup analysis on the dose of vitamin D used and the severity of the disease; 3) relatively small size of included studies (only 5 studies), publication bias assessment and funnel plot were not performed; 4) methods for vitamin D measurement were not standardized.

In conclusion, vitamin D supplementation did not significantly decrease the number or rate of exacerbation nor improve pulmonary function (FEV1 and FEV1/FVC) in COPD patients with VDD. However, a rise in the serum 25(OH)D concentration was observable in both short- and long-term treatment, reducing the adverse reactions of COPD patients to some extent. Still, more RCTs exploring the effect of vitamin D on COPD patients are needed for future studies.

## Data Availability

The datasets generated and analysed during the current study are not publicly available, but are available from the corresponding author on reasonable request.
