# Corrigendum to: Negligible effect of vitamin D supplementation on exacerbation in patients with chronic obstructive pulmonary disease: meta-analysis

**DOI:** 10.11613/BM.2024.021201

**Published:** 2024-04-15

**Authors:** Ye Hua, Ting Jiang, Jiangyi Feng, Mi Zou

**Affiliations:** 1 Department of general surgery, Chongqing Emergency Medical Center, Chongqing University Central Hospital, Chongqing, China; 2 Department of blood transfusion, Chongqing Emergency Medical Center, Chongqing University Central Hospital, Chongqing, China; 3 Respiratory department, The First branch of the first affiliated hospital of Chongqing Medical University, Chongqing, China

This is a correction of Biochem Med (Zagreb) 2023;33(3):030703. DOI: https://doi.org/10.11613/BM.2023.030703

Since the publication of the article, the authors have noticed that the statement “The first two authors contributed equally to this work.” is missing from the byline. The correct byline is presented above. We apologize to the authors for any inconvenience caused to the readers.

